# *Limosilactobacillus reuteri* DS0384 promotes intestinal epithelial maturation via the postbiotic effect in human intestinal organoids and infant mice

**DOI:** 10.1080/19490976.2022.2121580

**Published:** 2022-09-21

**Authors:** Hana Lee, Kwang Bo Jung, Ohman Kwon, Ye Seul Son, Eunho Choi, Won Dong Yu, Naeun Son, Jun Hyoung Jeon, Hana Jo, Haneol Yang, Yeong Rak Son, Chan-Seok Yun, Hyun-Soo Cho, Sang Kyu Kim, Dae-Soo Kim, Doo-Sang Park, Mi-Young Son

**Affiliations:** a Stem Cell Research Convergence Center, Korea Research Institute of Bioscience and Biotechnology (KRIBB), Daejeon, Republic of Korea; b KRIBB School of Bioscience, Korea University of Science and Technology (UST), Daejeon, Republic of Korea; c Korean Collection for Type Cultures, Biological Resource Center, KRIBB, Jeongeup, Republic of Korea; d Laboratory of Efficacy Research, Korea Ginseng Corp., Daejeon, Republic of Korea; e Digital Biotech Innovation Center, KRIBB, Daejeon, Republic of Korea

**Keywords:** Human intestinal organoid, intestinal development, epithelial maturation, gut microbiota, probiotic, postbiotic, *Limosilactobacillus reuteri*, N-carbamyl glutamic acid

## Abstract

Little is known about the modulatory capacity of the microbiota in early intestinal development. We examined various intestinal models that respond to gut microbial metabolites based on human pluripotent stem cell-derived human intestinal organoids (hIOs): physiologically relevant *in vitro* fetal-like intestine, intestinal stem cell, and intestinal disease models. We found that a newly isolated *Limosilactobacillus reuteri* strain DS0384 accelerated maturation of the fetal intestine using 3D hIO with immature fetal characteristics. Comparative metabolomic profiling analysis revealed that the secreted metabolite N-carbamyl glutamic acid (NCG) is involved in the beneficial effect of DS0384 cell-free supernatants on the intestinal maturation of hIOs. Experiments in an intestinal stem cell spheroid model and hIO-based intestinal inflamed model revealed that the cell-free supernatant from DS0384 comprising NCG promoted intestinal stem cell proliferation and was important for intestinal protection against cytokine-induced intestinal epithelial injury. The probiotic properties of DS0384 were also evaluated, including acid and bile tolerance and ability to adhere to human intestinal cells. Seven-day oral administration of DS0384 and cell-free supernatant promoted the intestinal development of newborn mice. Moreover, NCG exerted a protective effect on experimental colitis in mice. These results suggest that DS0384 is a useful agent for probiotic applications and therapeutic treatment for disorders of early gut development and for preventing intestinal barrier dysfunction.

## Introduction

Postnatal intestinal maturation plays an important role in the normal development and physiological function of the intestine, including establishment of the epithelial barrier and immune system as well as colonization and stabilization of the microbiota during the first two years of life.^1^ A key aspect of intestinal maturation is the development of barrier integrity, which is critical not only for nutrient absorption but also for preventing the entry of pathogenic bacteria and toxic substances.^2^ The initial colonization of gut microbiota and their interactions with the host can influence intestinal development and epithelial maturation by promoting the proliferation and differentiation of intestinal epithelial cells, vascularization, production of mucus, and maintenance of epithelial junctions.^3,4^ Failure of the intestine to mature normally has been implicated in the pathogenesis of neonatal intestinal diseases, such as necrotizing enterocolitis and early-onset inflammatory bowel disease (IBD). Very early onset IBD (VEO-IBD) is characterized by not only the common symptoms of adult IBD, such as rectal bleeding and diarrhea due to inflammation and intestinal epithelial disruption, but also growth failure.^5–7^

One strategy for reinforcing intestinal epithelial functions is administration of probiotics, which are defined as ‘live microorganisms that, when administered in adequate amounts, confer a health benefit on the host’.^8^ Increasing evidence has demonstrated the beneficial effects of certain probiotic strains in maintaining homeostasis of the intestinal environment, protecting barrier integrity, and improving its repair after damage.^9–11^ Lactic acid bacteria (LAB) are thought to be important probiotics in the healthy intestinal microbiota and establishing the *Lactobacillus* population early in life controls intestinal development and mucosal barrier functions.^12^ Early colonization with *Lactobacillus* spp. was shown to reduce the alkaline environment of the intestine, enhance antioxidant defense, increase mucus secretion, and protect the intestinal barrier by attenuating epithelial cell DNA damage.^13–15^ Recently, the focus of probiotic research has shifted from viable microbial cells toward postbiotics, which are the metabolites of probiotics, because of their potential health-promoting properties.^16^ Postbiotics produced from *Lactobacillus* spp. include a wide range of molecules, such as peptidoglycans, extracellular polysaccharides, secreted proteins, bacteriocins, and short-chain fatty acids, which mediate antimicrobial, anti-inflammatory, immunomodulatory, anti-tumor, and barrier-protective effects on the host.^17^ However, the bioactivities and detailed mechanistic properties of these postbiotics are complex and remain poorly understood.

Previous studies aimed at characterizing the interaction between intestinal microbiota and gut epithelium typically used tumor-derived intestinal cell lines, such as Caco2, HT-29, and HCT-8 cells, which do not fully mimic the human intestine. Recently, intestinal organoids containing intestinal stem cells (ISCs) and all differentiated cell types of the intestinal epithelium have been used to investigate the effects of commensal microbiota on the gut. However, most research on the commensal microbiota, such as *Lactobacillus* spp., has been based on primary murine enteroids and colonoids.^18^ It was only recently demonstrated that *Lactobacillus reuteri* D8 can improve intestinal epithelial proliferation and repair tumor necrosis factor (TNF)-induced epithelial damage using mouse intestinal organoids derived from the small intestine of C57BL/6 mice.^19^ However, the effects of *Lactobacillus* spp., including postbiotics (microbial metabolites) and their biologically active functions, on fetal and postnatal intestinal development have not been evaluated in a physiologically relevant human *in vitro* model or mouse model.

Unlike primary intestinal organoids derived from adult tissue, human pluripotent stem cell (hPSC)-derived intestinal organoids produced using a stepwise differentiation protocol have been shown to most closely resemble the immature human intestine during the fetal periods.^20,21^ We previously demonstrated that these immature human intestinal organoids (hIOs) can further develop into adult-like hIOs through an additional *in vitro* maturation process using interleukin-2.^22^ In this study, we examined the function of *Lactobacillus* spp. and evaluated its probiotic properties from the perspective of human intestinal maturation and functional development using the hPSC-derived hIO system, which is a physiologically relevant developmental and disease model of the human intestine. We identified a novel strain of *Limosilactobacillus reuteri* (previously known as *Lactobacillus reuteri*^23^), DS0384, isolated from the feces of a healthy infant, and showed that DS0384-derived biologically active metabolites have beneficial effects on intestinal maturation. The active metabolite of DS0384 induced to transit immature hIO to adult-like mature hIO, and the DS0384 strains and CFS improved neonatal development in the mouse intestine. Through metabolomic profiling, we identified N-carbamyl glutamic acid (NCG) in the cell-free supernatants (CFS) of DS0384, which effectively promoted intestinal development in hIO model. CFS from DS0384 also promoted ISC proliferation in an ISC spheroid model. Moreover, CFS from DS0384 containing NCG reduced the inflammatory phenotypes of an hIO-based intestinal inflamed model and exerted a protective effect on mice with dextran sodium sulfate (DSS)-induced colitis, highlighting the potential of DS0384 to relieve the pathological status of the human intestine.

## Results

### Cell-free supernatants from *L. reuteri* improve hIO maturation

The effects of metabolites produced by five probiotic strains (*Bifidobacterium longum. Lactobacillus gasseri, Lactobacillus crispatus, Lacticaseibacillus rhamnosus*, and *L. reuteri*) on intestinal maturation was determined using hPSC-derived hIO, a powerful tool for mimicking *in vitro* postnatal intestinal development. The surface area and number of crypt-like budding structures, which are phenotypic characteristics of mature and more differentiated hIOs, were most significantly increased in hIOs treated with CFS from *L. reuteri* (Supplementary Figure S1(a–c)). In addition, the CFS obtained from *L. reuteri* significantly increased the expression of mature intestine-specific markers, including an intestine-specific marker (*CDX2*), an ISC marker of the mature intestine (OLFM4), Paneth cell markers (*DEFA5* and *LYZ*), and mature intestinal differentiation markers (*KRT20, CREB3L3, DPP4, LCT, SLC5A1*, and *MUC13*) at the mRNA and protein levels (Supplementary Figure S1(a,d)).

**Figure 1.**
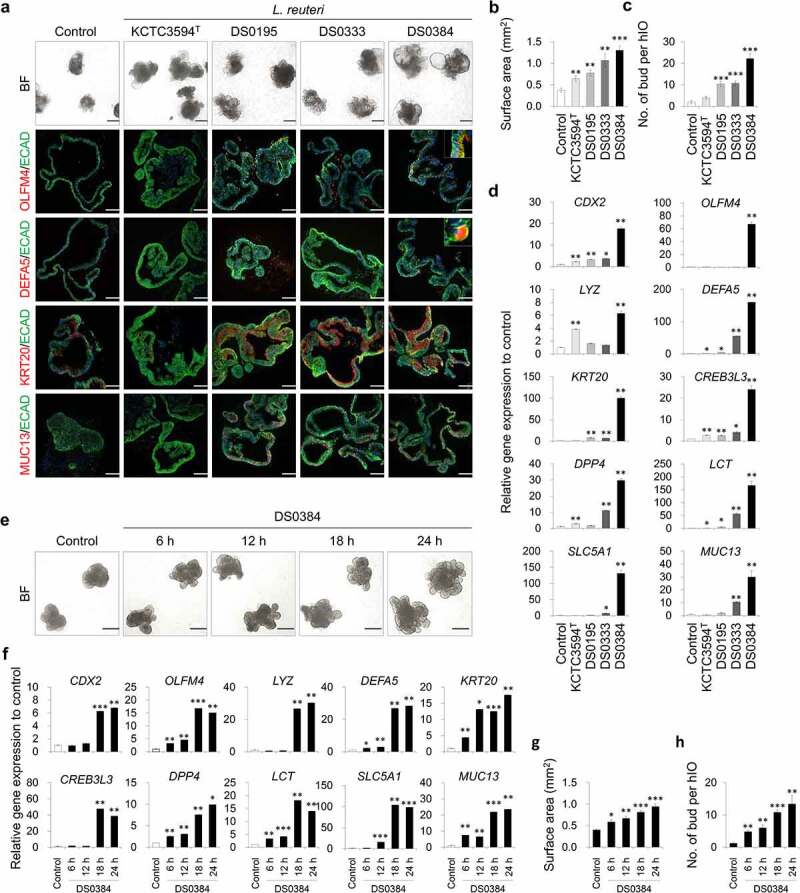
*Limosilactobacillus reuteri* DS0384 improved maturity of 3D hIO. (a) Representative images of morphologies and immunofluorescent staining in four *L. reuteri* strains: KCTC3594^T^, DS0195, DS0333, and DS0384. Black scale bars, 500 μm, white scale bars, 100 μm. Quantitative data of surface area (b) and number of budding structures of hIOs (c) treated with cell-free supernatants (CFS) from four *L. reuteri* strains. Data represent the means ± SEM (n = 10 4x fields from at least 10 organoids). (d) qPCR analysis of the intestine-specific and mature small intestine marker genes in control hIOs and hIOs treated with CFS from four *L. reuteri* strain. Data represent the means ± SEM (n = 3). (e) Representative images of morphologies of control hIOs after treatment with CFS harvested at different times from *L. reuteri* DS0384. (f) Relative expression levels of intestine-specific and mature intestine makers in hIOs by qPCR. Data represent the means ± SEM (n = 3). Quantitative data of surface area (g) and number of budding structures of hIOs (h) treated with CFS harvested at different times from *L. reuteri* DS0384. Data represent the means ± SEM (n = 10). *p < .05, **p < .01, ***p < .001 by two-tailed *t*-test.

To comprehensively investigate the intestinal maturation effect of *L. reuteri*, hIOs were treated with CFS from various *L. reuteri* strains, including *L. reuteri* type strain KCTC3594^T^ and newly isolated *L. reuteri* strains (DS0195, DS0333, DS0384). Overall differences were observed in the intestinal maturation-inducing capacity of the *L. reuteri* strains; DS0384 most significantly increased the surface area of hIOs (Figure 1(b), number of budding structures of hIOs (Figure 1(c), and expression of mature intestine-specific markers both at the mRNA and protein levels (Figure 1(a,d)). The hIO maturation effect of CFS from DS0384 was confirmed across hIOs differentiated from two independent human induced pluripotent stem cell (hiPSC) lines (Supplementary Figure S2). Furthermore, harvesting the CFS of DS0384 at different time points determined referring the growth curve of DS0384 supported this maturation effect (Supplementary Figure S3). Maturation of hIO was found to be promoted at later collection times (6, 12, 18, and 24 h) based on the significant increase in the expression of mature intestine-specific genes and changes in morphology (Figure 1(e–h)). These results suggest that active components are present in the CFS of DS0384.

### Whole-genome sequencing and characterization of probiotic *L. reuteri* strain DS0384

The *L. reuteri* DS0384 strain was isolated from the feces of a healthy infant. To determine its genomic characteristics, whole-genome sequencing was performed using PacBio RSII single-molecule real-time (SMRT) sequencing technology. The complete genome consisted of a single circular chromosome of 2,222,886 bp and circular plasmids of 20,351 bp with a 39.06% G + C content. A total of 2,258 genes was predicted in the genome of this strain. Of these, 2,191 were protein-coding genes, and 1,328 of these protein coding genes were assigned putative functions. The remaining proteins were annotated as hypothetical proteins. Whole-genome sequences were deposited under Bioproject PRJNA971146 and Biosample SAMN24255923, respectively. The GenBank accession numbers were CP090313 for a single chromosome and CP090314 for the plasmid. The genome-genome relatedness of *L. reuteri* DS0384 was also analyzed by calculating the average nucleotide identity (ANI) and constructing a phylogenomic tree of 31 genome sequences with fewer than 30 scaffolds among the genomes of *L. reuteri* strains from the GenBank/EMBL/DDBJ database. The whole genome of *L. reuteri* DS0384 showed ANI values of 93.8–99.9% with other *L. reuteri* strains (Supplementary Figure S4(a)). The highest ANI values were obtained with the SD2112 strain, which was isolated from breast milk; the complete genome consisted of a single circular chromosome of 2,264,399 bp and three circular plasmids.^24^ In total, 1,660 orthologous genes were shared between the DS0384 and SD2112 strains (Supplementary Figure S4(b)). A phylogenetic tree of the 31 *L. reuteri* strains was constructed based on amino acid alignments of 92 core genes using the maximum likelihood approach. This analysis revealed that *L. reuteri* DS0384 clustered with the SD2112 strain, which is frequently used as a food additive^25^ (Supplementary Figure S5).

### Postbiotic metabolites produced by *L. reuteri* DS0384 exert intestinal maturation effects on hIO

**Figure 2.**
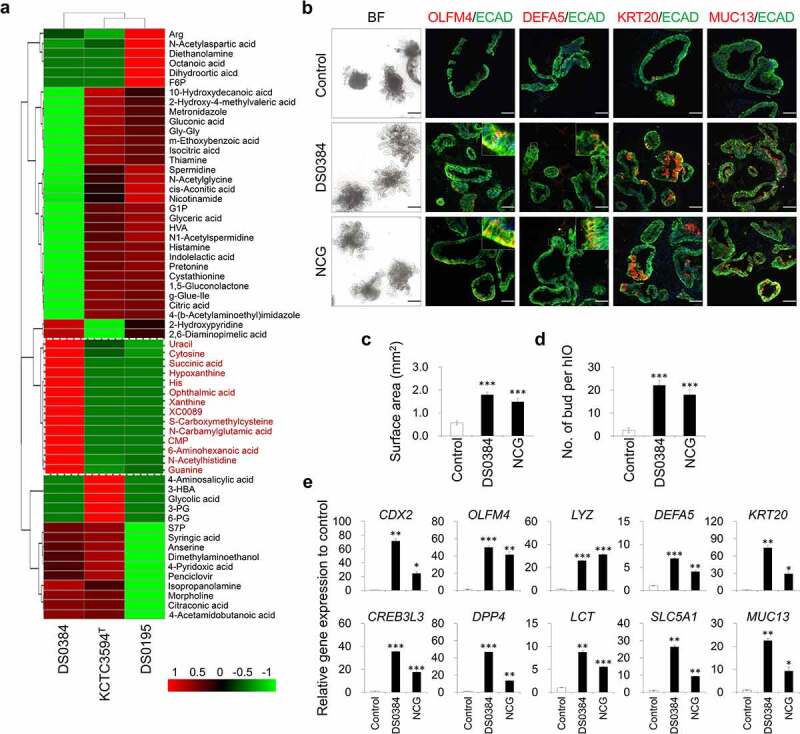
Intestinal maturation effect of postbiotic metabolite N-carbamyl glutamic acid produced by *L. reuteri* DS0384 on hIO. (a) Heatmap analysis for quantitative analysis of metabolite produced from *L. rueteri* strains, DS0384, KCTC3594^T^, DS0195. (b) Representative images of morphologies (bright field, BF) and immunofluorescent staining in control and cell-free supernatants (CFS) from DS0384-treated and N-carbamyl glutamic acid (NCG)-treated hIOs. Black scale bars, 500 μm, white scale bars, 100 μm. Quantitative data of surface area (c) and number of budding structures (d) of control, CFS from DS0384-treated, and NCG-treated hIOs. (e) qPCR analysis of the intestine-specific and mature intestine marker genes in control, CFS from DS0384-treated, and NCG-treated hIOs. Data represent the means ± SEM (n = 3) *p < .05, **p < .01 by two-tailed *t*-test.

From the capillary electrophoresis time-of-flight mass spectrometry (CE-TOFMS) measurement, 275 peaks (189 in cation and 86 in anion mode) were detected and annotated according to HMT’s standard library and Known-Unknown peak library. Supplementary Table S1 lists all 275 metabolites and their relative amounts in the samples. Among the target metabolites, 14 metabolites showed higher quantities in the CFS from DS0384 compared to that from the *L. reuteri* type strain KCTC3594^T^ and *L. reuteri* strain DS0195 (Figure 2(a)).

Next, we tested whether the bioactive metabolites involved in development were present in the CFS from DS0384. When monitoring the changes in hIOs by incubating the top seven differentially secreted metabolites, NCG was shown to have the greatest effects on intestinal maturation, as evidenced by increased budding numbers of hIOs and enlarged organoid morphologies (Figure 2(b–d)) and increased expression of intestinal maturation markers (Figure 2(b, e)). Although other metabolites, such as succinate, histidine, xanthine, cytosine monophosphate (CMP), 6-aminohexanoic acid (6-ACA), and *S*-carboxymethylcysteine (SCMC), also slightly increased the expression of intestinal maturation markers, their effects were considered as insignificant compared to those of NCG (Supplementary Figure S6).

### Metabolites derived from *L. reuteri* DS0384 increased proliferation of 3D InS^exp^

**Figure 3.**
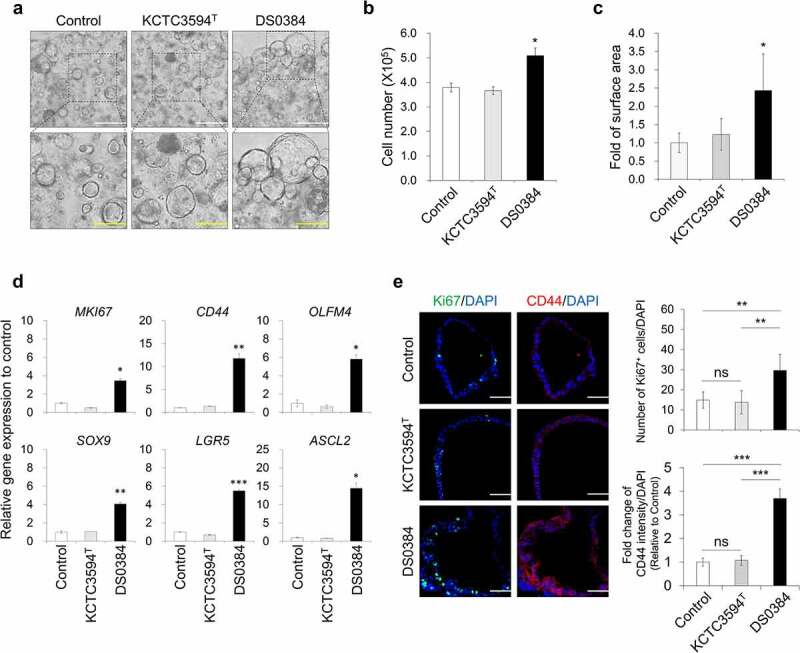
Intestinal stem cell proliferation effect of postbiotic metabolites produced by *L. reuteri* DS0384 on 3D InS^exp^. Effect of cell-free supernatants (CFS) from DS0384 and KCTC3594^T^ on InS^exp^ growth rate. (a) Bright field (BF) images of InS^exp^. White scale bars, 500 μm; yellow scale bars, 250 μm. The counted cell numbers (b) and quantification of surface area (c) in InS^exp^ treated with CFS from DS0384 or KCTC3594^T^. Data represent the mean ± SEM (n = 3). (d) qPCR analysis of intestinal stem cell marker genes in control hIOs and hIOs treated with CFS from DS0384 and KCTC3594^T^. Data represent the mean ± SEM (n = 3). (e) Immunofluorescence imaging and quantification analysis of proliferation (Ki67), intestinal stem cell markers (CD44), and nuclei (DAPI) in 3D InS^exp^. Scale bars, 50 μm. Data represent the mean ± SEM (n = 3). *p < .05, **p < .01, ***p < .001 by two-tailed *t-*test.

To assess the effects of metabolites derived from DS0384 on ISCs, which contribute to intestinal regeneration, we treated 3D InS^exp^ comprised of ISCs and intestinal progenitors with the CFS from *L. reuteri*. Metabolites contained in the CFS derived from DS0384 significantly increased the size of InS^exp^, viable cell number (Figure 3(a–c)), and expression levels of ISC marker genes, such as *CD44, OLFM4, SOX9, LGR5*, and *ASCL2* (Figure 3(d)). Ki67(+) proliferating cells and CD44(+) ISCs were significantly increased in the InS^exp^ following treatment with CFS derived from DS0384 compared to the control and CFS derived from KCTC3594^T^ (Figure 3(e)). These results indicate that metabolites derived from DS0384 exert ISC proliferation-stimulating activity during intestinal epithelial regeneration.

### Metabolites derived from *L. reuteri* DS0384 protect hIO against IFNγ/TNFα-induced inflammation

**Figure 4.**
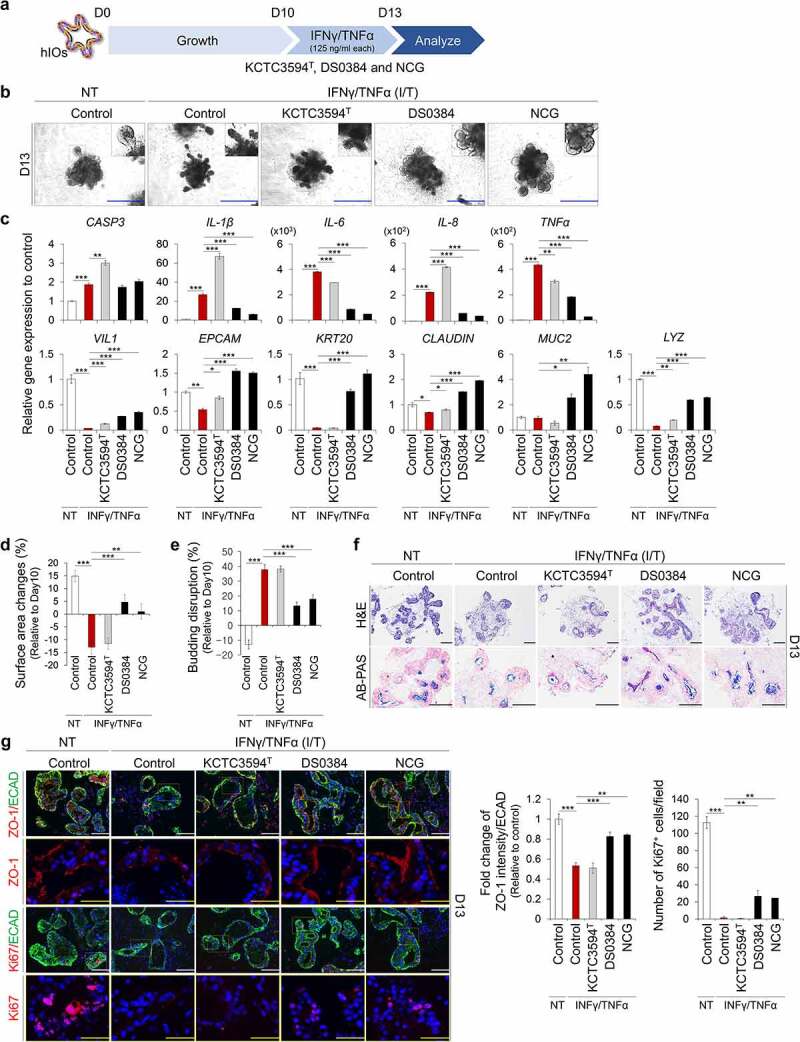
Protective effects of postbiotic metabolites produced by *L. reuteri* DS0384 on IFNγ/TNFα-induced inflamed hIO. (a) Schematic diagram of experimental design. (b) Morphology of control and IFNγ/TNFα-induced inflamed hIOs following treatment with cell-free supernatants (CFS) from *L. reuteri* KCTC3594^T^ and DS0384, and metabolite NCG. Scale bar, 1 mm. (c) qPCR analysis of apoptosis, inflammatory, intestine-specific, and intestinal barrier markers in inflamed hIOs and control hIOs (n > 10) treated with CFS from *L.reuteri* KCTC3594^T^ and DS0384, and metabolite NCG. (d) Percentages of surface area changes. Data represent the means ± SEM (n > 10). (e) Percentages of budding disruption. Data represent the means ± SEM (n > 10). (f) Histological analysis of control and inflamed hIOs following treatment with CFS from *L. reuteri* DS0384. Scale bar, 200 μm. (g) Immunofluorescence analysis of intestinal barrier (zonula occludens-1, ZO-1) and proliferating marker (Ki67). White scale bar, 125 μm, yellow scale bar, 50 μm. Fold-change in intestinal barrier (ZO-1) intensity to intestinal epithelial marker (ECAD) (middle, n > 5). Number of Ki67+ cells per field (right), (n = 5–10 10x fields from at least 10 organoids). Data are the mean ± SEM. *p < .05, **p < .01, ***p < .001 by two-tailed *t*-test.

We investigated whether metabolites derived from *L. reuteri* could protect against pathological conditions such as interferon γ (IFNγ)/TNFα-induced intestinal inflammation and barrier damage (Figure 4(a)). After 72 h of treatment with IFNγ/TNFα, the budding structures had decreased in size, a cellular phenotype associated with intestinal damage (Figure 4(b)). The expression of inflammatory cytokines, such as IL-1β, IL-6, IL-8, and TNFα, was significantly increased (Figure 4(c)), indicating that an *in vitro* inflamed human intestinal model was successfully established from hIOs. Compared to the IFNγ/TNFα treated group, treatment with the DS0384-derived CFS and their metabolite NCG maintained the normal intestinal epithelial structure of hIOs, as evidenced by reduced IFNγ/TNF-α-induced surface area contraction and budding disruption (Figure 4(d,e)). Consistently, the expression of inflammatory cytokines was significantly reduced by treatment with CFS derived from DS0384 and their metabolite NCG, compared to the control in inflamed hIOs (Figure 4(c)). Histological analysis *via* Alcian blue-periodic acid-Schiff (AB-PAS) staining showed that treatment with CFS derived from DS0384 and NCG decreased organoid destruction and preserved the mucus layers on hIOs (Figure 4(f)). In contrast, CFS derived from KCTC3594^T^ type strain had no protective effects on inflamed hIOs (Figure 4(b–f)). Moreover, treatment with CFS derived from DS0384 and NCG recovered the expression and localization of zonula occludens-1, which is associated with intestinal epithelial barrier integrity near the control level, and Ki67(+) proliferating cells were significantly increased compared to inflamed hIOs (Figure 4(g)).

### *Limosilactobacillus reuteri* DS0384 characteristics as a probiotics strain

**Figure 5.**
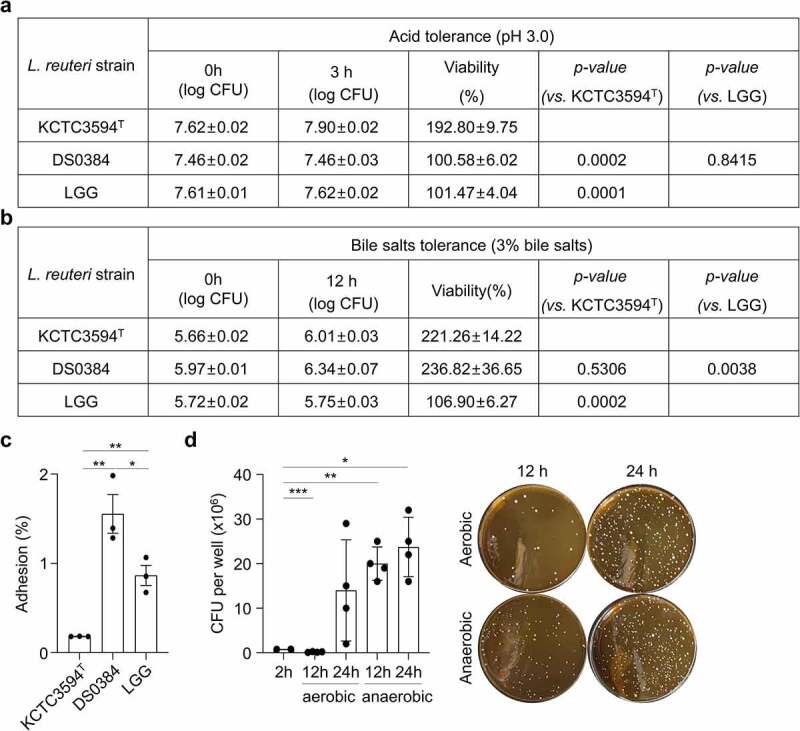
Cell adhesion and proliferation properties of *L. reuteri* DS0384. (a) Viability of *L. reuteri* DS0384, KCTC3594^T^, and LGG at pH 3.0. (b) Effect of bile salts on *L. reuteri* DS0384, KCTC3594^T^, and LGG. (c) Average adhesion of *L. reuteri* DS0384, KCTC3594^T^, and LGG to Caco-2 cells. Data are the mean ± SEM (n = 3). (d) Survival of DS0384 in an hIEC model under aerobic and anaerobic conditions. Data represent the mean ± SEM (n > 3). *p < .05, **p < .01, ***p < .001 by two-tailed *t*-test.

One of the criteria of a useful probiotic candidate is high survival in the gastrointestinal tract (GIT). We compared the viability of *L. reuteri* DS0384 and KCTC3594^T^ in an acidic environment, and found that DS0384 displayed tolerance to a pH as low as 3.0, with a viability of 100% (Figure 5(a)). DS0384 showed a high survival rate in 3% bile salts, with a viability of over 200% (Figure 5(b)). In a multiple-step *in vitro* digestion model, DS0384 showed 24.9% viability after 2 h treatment with salvia and gastric juice; this was higher than that of KCTC3594^T^ (9.82%) and *L. rhamnosus GG* (LGG) (17.11%). After 2 h treatment with digestive juice containing salvia, gastric juice, duodenal juice, bile juice, and NaHCO_3_, all LAB strains exhibited similar survival rates (Supplementary Table S2). In bacterial adhesion assays 1.55% of DS0384 cells adhered to Caco-2 cell monolayers, representing approximately 8.51-fold and 1.80-fold increases in adherence compared to that of the reference type strain KCTC3594^T^ (0.18%) and of LGG (0.87%), respectively (Figure 5(c)). DS0384 can survive and grow for 24 h in human intestinal epithelium cells (hIECs), forming a model of normal intestinal physiology that includes various intestinal cell types, as well as functionality and expression of mucin-related genes/proteins for bacterial adhesion^26^ in both aerobic and anaerobic conditions (Figure 5(d)). DS0384 exhibited comparable survival to the commercial probiotic LGG while passing through the GI tract and grew well in the small intestinal cell model for at least 24 h, suggesting potential probiotic effects.

### Intestinal maturation-stimulating effect of *L. reuteri* DS0384 in infant mice

**Figure 6.**
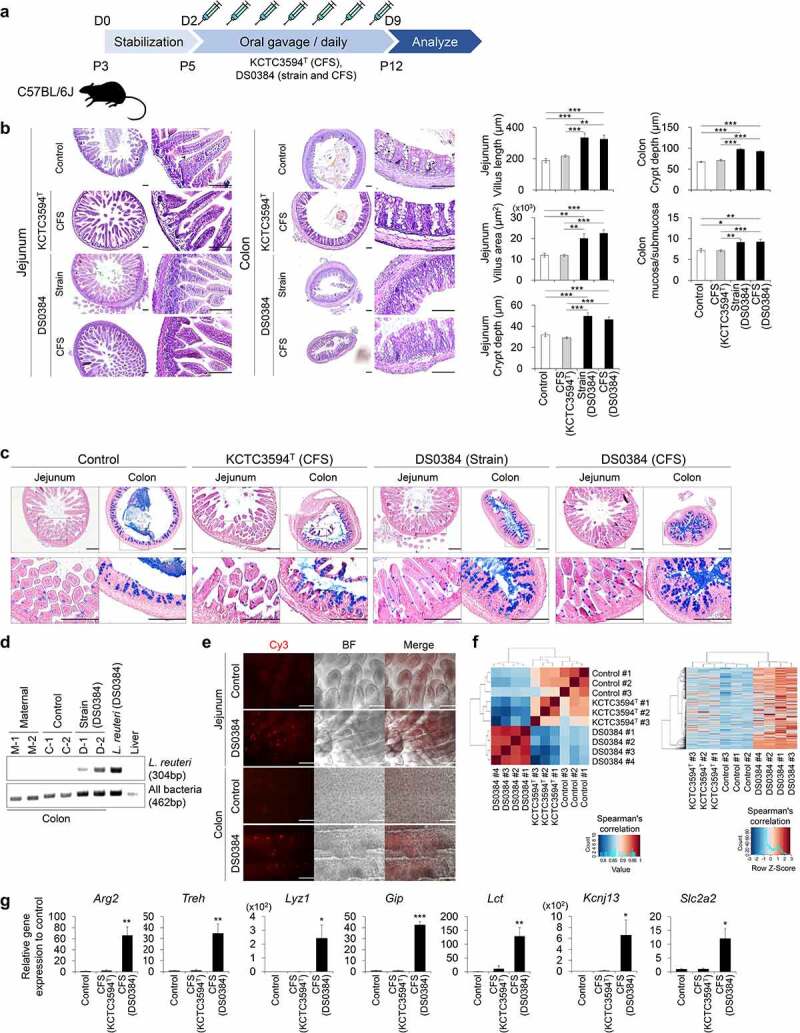
*Limosilactobacillus reuteri* DS0384 accelerates small intestine (jejunum) and colon maturation in infant mice. (a) Schematic diagram of experimental design in infant mice at postnatal day 5 (P5). (b) Hematoxylin and eosin staining of jejunum and colon following treatment with KCTC3594^T^ (CFS) and DS0384 (strain and CFS) (left). Histological analysis of villus length, area, crypt depth in jejunum, colon crypt depth, and ratio mucosa to submucosa (right). Black scale bar, 100 μm (bottom, n > 7 per group). Data represent the mean ± SEM. *p < .05, **p < .01, ***p < .001 by one-way ANOVA with a post hoc analysis and *t*-test. (c) AB-PAS staining for mucus layer and mucus producing goblet cells at postnatal day 12. Scale bar, 200 μm (bottom, n > 3 per group). (d) Representative PCR product image from *L. reuteri* and 16S rDNA PCR assay in the colon, live *L. reuteri* (DS0384), and liver. The experimental groups of mice colon were maternal, control (PBS), and DS0384 (strain) (n = 2 per group). (e) FISH analysis of *L. reuteri* in the colon of control (PBS) and DS0384 (strain) groups. Colonized *L. reuteri* were detected by FISH with Cy3-labeled, rRNA-targeted oligonucleotide probes. Scale bar, 125 μm. (f) Hierarchical clustering of the intestine-related gene set from the RNA-seq of intestinal tissue following oral administration of control (PBS) treatment or CFS from KCTC3594^T^ or DS0384. (g) qPCR analysis of the mature intestine-specific markers in tissue administered with PBS or CFS from KCTC3594^T^ or DS0384. Data represent the mean ± SEM (n ≥ 3). *p < .05, **p < .01, ***p < .001 by two-tailed *t*-test.

To confirm the intestinal maturation effect and potential use of DS0384, the DS0384 strain and CFS derived from DS0384 were orally administered to infant mice (Figure 6(a)). On day 5 after birth, the mice were orally administered PBS (control), CFS derived from KCTC3594^T^, CFS derived from *L. reuteri* DS0384, or live *L. reuteri* DS0384 for 7 d, and intestinal growth was evaluated. Mice fed with either live DS0384 or its CFS showed a significant increase in the villus length, villi area, and jejunum crypt depth at postnatal day 12 (Figure 6(b)). Consistent with these results, the crypt depth in the colon and the ratio of mucosa to submucosa were increased (Figure 6(b)). In contrast, feeding CFS derived from KCTC3594^T^ to neonatal mice had no significant effect on the growth and maturation of the small and large intestines during development compared to PBS treatment. To confirm the functional maturation of the GIT, we performed AB-PAS staining of the mucus layer and mucus-secreting goblet cells from the jejunum and colon (Figure 6(c)). Oral administration of live DS0384 or its CFS significantly enhanced mucus secretion, and differentiation of mucus-producing cells (goblet cells) differed from that in the control group (Figure 6(c)). AB-PAS-stained goblet cells in the jejunum of mice fed DS0384-derived CFS were already present along the villi and crypt-villi junction compartment at postnatal day 12, corresponding to those of control mice at postnatal day 14 (Supplementary Figure S7). To investigate whether *L. reuteri* DS0384 directly affected intestinal development due to its existence in intestinal tissues, PCR and fluorescence *in situ* hybridization (FISH) assays were performed with an *L. reuteri*-specific primer and probe, respectively (Figure 6(d, e)). *Limosilactobacillus reuteri*–specific PCR products were identified in the postnatal mice group fed live DS0384 for 3 days when compared with maternal and PBS control groups. It was also observed in the DS0384 strain as a positive control but was not in the liver of C57BL/6 J mice, representing a negative control (Figure 6(d)). The FISH assay was positive, showing that DS0384 detected colon tissue (Figure 6(e)). Next, we performed RNA sequencing (RNA-seq) analyses of intestinal tissues after mice were fed PBS (control), CFS from DS0384, or CFS from KCTC3594^T^ for 3 d to assess transcriptome-level changes. Hierarchical cluster analysis of the RNA-seq dataset showed upregulation of genes related to intestinal differentiation and maturation in the intestine of mice administered CFS from DS0384 compared with those in mice provided PBS or CFS from KCTC3594^T^ (Figure 6(f) and Supplementary Table S3). In association with RNA-seq results, intestinal development-related genes, such as *Arg2, Treh, Lyz1, Gip, Lct, Kcnj13*, and *Slc2a2*, were significantly increased in the intestine of mice administered CFS from DS0384 (Figure 6(g).

### NCG protects against DSS-induced colitis in a mouse model

**Figure 7.**
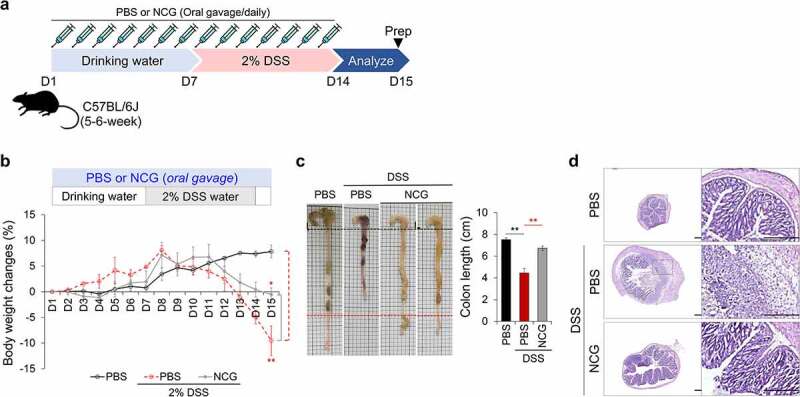
Protective effects of NCG in a DSS-induced colitis mouse model. (a) Schematic diagram of experimental design in a colitis mouse model. The experimental groups were control, DSS+PBS, and DSS+NCG (100 mM) (n = 3 per group). (b) Relative body weight change over 15 d. (c) Representative photographs of the experimental colon (left) and graph of colon length (right). (d) H&E staining for histopathological analysis in the colon. Data represent the mean ± SEM. *p < .05, **p < .01 by one-way ANOVA with a post hoc analysis and *t*-test.

We examined the intestinal protective efficacy of NCG in a mouse colitis model. Mice received 100 mM NCG in 200 μL of PBS via oral gavage for 7 d, and then colitis was induced by adding DSS (2%) to drinking water for an additional 7 d. The DSS control group was co-administered PBS, and the NCG group received daily co-administration of NCG with DSS throughout the experiment (Figure 7(a)). Four days after exposure to DSS, the rate of weight loss was slower in the NCG group than in the DSS control group (Figure 7(b)). Compared to the control group, the NCG group exhibited fewer diarrhea and bloody stool reactions. The colon length of the NCG group was maintained at the control level, and the intestinal feces showed a pellet shape (Figure 7(c)). NCG administration was associated with fewer histological alterations caused by DSS (Figure 7(d)). These results suggest that NCG exerted a protective effect on DSS-induced colitis.

## Discussion

We investigated the effects of various LAB strains on intestinal tract development by examining the maturation status of hIO. The advantage of this culture system is that hPSC-derived immature hIOs can be transitioned into adult-like mature hIOs when they receive the proper maturation signals, thereby providing an ideal *in vitro* intestinal development model. A fundamental step during which the GIT acquires its function is intestinal epithelial maturation, which is marked by the emergence of various intestinal cell types, morphogenesis with crypt-villus characteristics, and improved structural integrity. For example, specifically in the mature intestine, ISCs express OLFM4 and Paneth cells secrete α-defensin (DEFA4) for host defense.^20^ The expression of cytokeratin KRT20, a member of the intermediate filament family, and functional goblet marker MUC13 indicates improved intestinal epithelial integrity and functionality.^27,28^ hPSC-derived hIOs are an excellent *in vitro* model for studying the intestinal maturation status. Additionally, when 3D hIOs are matured *in vitro*, they show a large size with complex budding structures, resembling the crypt-villus structure in the intestine.

To identify LAB associated with epithelial maturation during early intestinal development, we selected strains thought to be transmitted from the mother to newborns and infants through birth and lactation. The functions of postbiotic metabolites in early human intestinal development were analyzed using a physiologically compatible 3D human organoid system to identify novel LAB for treating disorders of early gut development and preventing intestinal barrier dysfunction. *Lactobacillus crispatus* has important beneficial effects on the vaginal microbiome of the mother, and *L. gasseri* is a representative strain found in breast milk.^29^ *Bifidobacterium longum, L. reuteri*, and *Lacticaseibacillus rhamnosus* can be directly isolated from the feces of newborns and are thought to play an important role in the composition and health of intestinal microbiomes in early intestinal development.^30,31^ Among the tested LAB strains, hIOs treated with CFS derived from *L. reuteri* DS0384 showed distinctive hIO differentiation and maturation patterns. The hIO maturation effect caused by CFS from DS0384 was confirmed across hIOs differentiated from the ESC line, and in two independent hiPSC lines, suggesting limited impact of batch-to-batch variability^32,33^(Supplementary Figure S2). The intestinal development effect of the DS0384 strain and CFS from DS0384 in postnatal mice was shown in both the small and large intestine, and it was confirmed that the mucus secretion related to digestion was increased (Figure 6(a-c)). In particular, DS0384 strain exists in the intestine of bacterial strain-fed mice after 3 days of gavage (Figure 6(d,e)), indicating that the possibility of intestinal colonization. *Limosilactobacillus reuteri* is an important resident microflora in the intestines of all vertebrates, including mammals.^34^ DS0333 showed a moderate effect on hIO maturation, both on the morphology and marker expression, compared to DS0384, whereas DS0195 and KCTC3594^T^ showed a weaker effect on hIO maturation (Figure 1(a–d)), indicating that the intestinal maturation effect of the postbiotic metabolites of *L. reuteri* is strain-dependent rather than a common characteristic of the respective species. In addition, the active metabolites were detected continually throughout the growth phase and stably concentrated in the culture broth, showing that the intestinal maturation effect of the CFS from DS0384 gradually increased from the exponential to the late stationary phase of growth (Figure 1(e-h) and Supplementary Figure S3).

Comparison of the metabolome profiles of *L. reuteri* strains revealed 14 differentially expressed metabolites in DS0384 compared with those in DS0195 and KCTC3594^T^ (Figure 3(a)). These metabolites are related to glutamine metabolism and the urea cycle (spermidine, ophthalmic acid, histidine) and purine or pyrimidine metabolism (xanthine, hypoxanthine, guanine, CMP, uracil, cytosine) and were not identified on the pathway map (6-ACA, NCG, SCMC, XC0089). Among them, NCG was identified as a major effector of hIO maturation (Figure 2(b–e)). hIO maturation was improved with CFS from DS0384 strain at longer cultivation times (Figure 1(e-h)), demonstrating that these metabolites are produced directly by DS0384. NCG is a metabolically stable analog of N-acetylglutamate that activates carbamyl phosphate synthetase-1, the first enzyme involved in the urea cycle.^35^ Moreover, NCG is synthesized by amino acid carbamylation, a non-enzymatic, single-step spontaneous reaction between the primary amine L-glutamic acid and isocyanate in an aqueous media.^36^ The genetic and molecular mechanisms by which *L. reuteri* enhances NCG synthesis remain unknown. We suggest that the increase in NCG levels occurs due to the enhanced production of precursors, such as isocyanic acid and glutamic acid. Isocyanic acid is produced by thermal decomposition of urea, and *Lactobacilli* strains are known to increase urea levels by the activation of ammonia assimilation.^37,38^ Therefore, it is possible that the *L. reuteri* increased the isocyanic acid as an intermediate metabolite of ureagenesis, and the amount of NCG was increased by carbamylation of L-glutamic acid with isocyanic acid. However, we did not identify a homologous putative carbamyl transferase in the whole-genome data, which may produce NCG from a hypothetical precursor, such as glutamate, N-acetylglutamate, or its relatives. Future studies should focus on elucidating the exact mechanism of NCG production by *L. reuteri*. NCG is not toxic to animals and infants and readily enters cells and mitochondria.^39,40^ Increasing evidence indicates that NCG plays a crucial role in the metabolism of the endogenous Arginine and Arginine families of amino acids to produce nitric oxide and polyamines *via* urea circulation.^41^ NCG is used as an important feed additive in dairy cows, beef cattle, neonatal pigs, and mutton sheep to increase milk production, enhance the pregnancy rate, reduce morbidity and mortality, and increase birth weight and growth rate.^42–46^ However, natural synthesis of NCG has not been reported. Therefore, the beneficial gut microbe *L. reuteri* DS0384, which produces NCG, may be useful for improving the health of people and livestock.

Traditional intestinal inflammation models, such as immortalized intestinal human cell lines under monolayer culture conditions cannot mimic the complex interactions occurring *in vivo*.^47^ A recent study demonstrated that intestinal organoids derived from inflamed tissues of patients with active IBD had distinct phenotypes with a smaller size, reduced budding numbers, increased cell death, poor polarization of epithelial cells, and reduced expression patterns of tight junction proteins.^48^ However, these adult-tissue-derived intestinal organoids exhibited patient-specific characteristics and low reproducibility between patient samples. A recent study showed that acute IFNγ treatment resulted in minimal disruption of junctional complexes in hPSC-derived hIOs.^49^ In this study, in the inflamed intestinal epithelial model, which was induced by exposing hPSC-derived hIOs to IFNγ/TNFα, showed upregulation of inflammatory cytokines such as IL-1β, IL-6, IL-8, and TNFα, which contribute to IBD development. We also observed destruction of the budding structures and an altered distribution of tight junction molecules (Figure 4(a–g)). Moreover, the CFS derived from *L. reuteri* DS0384 protected against morphological damage and reduced the expression of inflammatory cytokines, suggesting its utility as a probiotic. Additionally, NCG, a metabolite of *L. reuteri* DS0384, exhibited a protective effect on hIOs (Figure 4) and relieved symptoms in a mouse model of DSS-induced colitis (Figure 7).

*Limosilactobacillus reuteri*, which has been detected in the breast milk of Finnish women, is considered as a native colonizer of the GIT of humans and animals.^12^ Colonization of LAB at an early age can protect infants against atopic allergy.^50^ In recent clinical trials, the probiotic formulation VSL#3, containing four lactobacilli, three bifidobacteria, and one streptococci strain, exerted a beneficial effect on intestinal disease states, such as gastric ulcer,^51^ ulcerative colitis,^52,53^ irritable bowel syndrome,^54,55^ and microscopic colitis^56^ in patients. Herein, we showed that NCG produced by *L. reuteri* DS0384 induced intestinal maturation using a hIO similar to the neonatal intestine and protected against cytokine-induced intestinal epithelial injury in an inflamed hIO model. These findings indicate that the DS0384 strain and its postbiotic substances can be administered to infants with an immature intestine and to patients with inflamed intestines.

In conclusion, we identified and characterized a novel probiotic strain, *L. reuteri* strain DS0384. This strain showed postbiotic potential, as it promoted intestinal epithelial maturation and protected the intestinal epithelium from IFNγ/TNFα-induced injury in hPSC-derived hIO models. Further *in vivo* studies demonstrated that the DS0384 strain and its CFS improved intestinal development and epithelial maturation in infant mice. Hence, the reliable and robust hPSC-derived hIO system is a versatile intestinal experimental platform for investigating intestinal maturation and the disease status. Our research provides insight into the mechanism of probiotic action and a foundation for promoting intestinal development in newborns and infants as well as protecting against intestinal barrier dysfunction-related diseases.

## Materials and methods

### Generation of 3D hIOs from hPSCs

This study was approved by the Korean Public IRB (IRB number: P01-201409-ES-01-09, P01-201609-31-002). The H9 hESC line (WiCell Research Institute, Madison, WI, USA) and two independently derived hiPSC lines were routinely cultured as described previously.^22^ The 3D hIOs were differentiated as described previously.^22^ hPSCs were induced into definitive endodermal cells by treatment with 100 ng/mL Activin A (R&D Systems, Minneapolis, MN, USA) for 3 d in RPMI 1640 (Thermo Fisher Scientific, Waltham, MA, USA) and increasing concentrations of fetal bovine serum (FBS, 0%, 0.2%, 2% Thermo Fisher Scientific). To induce differentiation into hindgut (HG) stage cells, definitive endodermal cells were treated with 500 μg/mL FGF4 (R&D Systems) and 3 μM CHIR 99021 (Tocris Bioscience, Minneapolis, MN, USA) in RPMI 1640 medium containing 2% FBS for 4 d. Following HG induction, 3D HG spheroids spontaneously formed. 3D HG spheroids were placed in Matrigel (BD Biosciences, Franklin Lakes, NJ, USA) and cultured in hIO medium containing 100 µg/mL EGF (R&D Systems), 500 µg/mL R-spondin1 (R&D Systems), 100 µg/mL Noggin (R&D Systems), and 1X B27 supplement in advanced DMEM F12 (Thermo Fisher Scientific).

### Isolation of LAB strains

The LAB strains used in this study were isolated from infant feces, human milk, and vaginal mucosa of women and the small intestine of swine (Supplementary Table S4). The strains were isolated and identified as described previously.^57^ Briefly, serially diluted samples were spread onto Man, Rogosa, and Sharpe (MRS, BD Biosciences) agar plates and incubated in an anaerobic chamber (Coy Laboratory Products, Grass Lake, MI, USA) in the presence of 5% H_2_, 10% CO_2_, and 85% N_2_ at 37°C. After 2–3 d, a single ivory colony was obtained and identified by 16S rRNA sequencing. The isolated strains were deposited in the Bio R&D Product program (https://biorp.kribb.re.kr/) in Korea. The type strain was obtained from the Korean Collection for Type Cultures (KCTC, Daejeon, Korea). The growth of DS0384 strains was measured using a UV/VIS spectrophotometer (Optizen POP, Mecasys Co. Ltd, Korea) at 600 nm every 2 h after inoculation of 50 mL fresh MRS media with 1% seed culture (v/v). The colony forming units (CFU) were counted for each sample using the plate counting method.^58^

### Preparation of CFS and hIO treatment

The bacterial strains were cultivated in MRS broth under anaerobic conditions at 37°C for 24 h until reaching the stationary phase; *Bifidobacterium longum* was cultivated for 36 h under the same conditions. To investigate the hIO growth–promoting effect of the CFS according to the culture period of the DS0384 strain, the bacterial cultures were collected at 6, 12, 18, and 24 h after inoculation, then centrifuged at 3,000 × *g* for 10 min. The resulting CFS was heated at 65°C for 30 min and filtered through 0.22-µm syringe filter. The CFS was transferred to a new tube and stored at −80°C until use. hIOs were treated with CFS diluted with hIO culture medium at a ratio of 1:100 for two passages. The concentration of CFS used had no toxic effect on hIOs.

### Isolation and culture of 3D InS^exp^ derived from hIOs

The 3D InS^exp^ derived from 3D hIOs was generated and maintained as previously reported.^26^ The culture medium was replaced every other day. For passaging, InS^exp^ was harvested from the Matrigel dome by trypsin-EDTA (TE) treatment and pipetting with a 1000-µL pipette. The InS^exp^ was collected in a conical tube and then dissociated in a water bath at 37°C for 5 min. Basal medium was added to dilute TE up to 10 mL, and then the culture was centrifuged at 1500 rpm and 4°C for 5 min. The cell pellet was resuspended in Matrigel. The hIO-Matrigel mixture was plated into a four-well culture plate and incubated at 37°C in a CO_2_ incubator for more than 30 min to solidify the Matrigel, followed by addition of InS^exp^ growth medium supplemented with 10 μM of Y-27632 (Tocris) and 1 μM Jagged-1 (AnaSpec, Fremont, CA, USA) for the first 2 d. This medium was replaced with InS^exp^ culture medium, which was then replaced every other day. In order to minimize the size variation of organoids, we used the same initial number of cells (approx. 0.5 × 10^5^ cells/dome) by trypsinized 3D InS^exp^ and filtering through a 40 μm nylon mesh. To detect the effect of metabolites derived from *L. reuteri*, InS^exp^ cells were cultured in medium containing 1:100 diluted CFS, and size changes of InS^exp^ were recorded every 2 d in four repeated experiments. To calculate the cell surface area, the cell diameter was measured using ImageJ software (NIH, Bethesda, MD, USA).

### Generation of inflamed hIOs and treatment

hIOs (p2-3) were cultured for 10 d prior to IFNγ/TNFα treatment. IFNγ/TNFα (125 ng/mL each) was added for 72 h to generate inflamed hIOs. To examine the effect of *L. reuteri*-derived metabolites on inflamed hIOs, hIOs were co-cultured with CFS (1:100 diluted) and INFγ/TNFα for 72 h. To verify the effect of *L. reuteri*-derived metabolites, hIOs were co-cultured with CFS (1:100) and 1 mM NCG in the medium for 8 d. For phenotypic analysis, ImageJ software was used to measure the surface area of the intestinal organoids and budding disruption.

### Cell counting

InS^exp^ cells were dissociated into single cells by treatment with 0.25% trypsin-EDTA for 10 min at 37°C. The dissociated cells were diluted with advanced DMEM/F12 basal medium and centrifuged for 5 min at 1500 rpm. Single cells were resuspended in InS^exp^ growth medium and stained with trypan blue. The cells were counted using a Countess 3 Automated Cell Counter (Thermo Fisher Scientific).

### Preparation and application of metabolites

Succinic acid, histidine, xanthine, CMP, 6-ACA, NCG, and SCMC were reconstituted according to the manufacturer’s instructions (Sigma-Aldrich, St. Louis, MO, USA). Succinic acid (1 mM), histidine (100 µM), xanthine (14 µM), CMP (1 mg/mL), 6-ACA (30 µM), NCG (1 mM), and SCMC (10 µM) were added to the hIO culture medium, and the medium was changed every other day. The hIOs were treated with the metabolites for two passages.

### Whole-genome sequencing and analysis

Whole-genome sequencing of DS0384 was performed using PacBio RS II (Pacific Biosciences, Menlo Park, CA, USA) SMRT sequencing technology. A standard PacBio library with an average of 20-kb inserts was prepared and sequenced, yielding >286× average genome coverage. *De novo* assembly of the 81,092 subreads with 9,687 nucleotides on average (785,607,610 bp in total) was conducted using the hierarchical genome-assembly process pipeline of SMRT Analysis v2.3.0. To correct sequencing errors that can occur at both ends of a contig, the SMRT resequencing protocol was performed with an assembly in which the first half of the contig was switched with the second half. Protein-coding genes were predicted using Prodigal v.2.6.3. Ribosomal RNA and transfer RNA, and miscellaneous features were predicted using the CRISPR recognition tool Rfam v12.0.^59^ CRISPR loci. ANI values were calculated using an online ANI calculator.^60^ Phylogenomic analysis was performed using 92 bacterial core genes based on the up-to-date bacterial core gene tool (https://www.ezbiocloud.net/tools/ubcg) against 31 *L. reuteri* strains derived from the GenBank/EMBL/DDBJ database. *Limosilactobacillus fermentum* NBRC3959 was used as an outgroup. Representative genes were selected based on 1429 complete genome sequences, covering 28 phyla and providing a set of genes present in most of the genomes or highly conserved single-copy genes.^61^

### Metabolome profiles of *Lactobacillus* culture medium analyzed using CE-TOFMS

To extract ionic metabolites, 80 µL of cell-free supernatants were mixed with 20 μL of Milli-Q water containing internal standards (1 mM). Metabolome analysis of three *L. reuteri* strains (DS0384, KCTC3594^T^, and DS0195) was conducted using the HMT Basic Scan package using CE-TOFMS according to previously described methods.^62,63^ Briefly, CE-TOFMS analysis was performed using an Agilent CE capillary electrophoresis system equipped with an Agilent 6210 time-of-flight mass spectrometer (Agilent Technologies, Santa Clara, CA, USA). The systems were controlled by Agilent G2201AA ChemStation software version B.03.01 (Agilent Technologies) and connected by a fused silica capillary (50μm i.d.×80cm total length) with commercial electrophoresis buffer (H3301-1001 and I3302-1023 for cation and anion analyses, respectively, HMT) as the electrolyte. The spectrometer was scanned from m/z 50 to 1,000, and peaks were extracted using MasterHands, automatic integration software (Keio University, Tsuruoka, Yamagata, Japan) to obtain peak information including the *m/z*, peak area, and migration time.^64^ Signal peaks corresponding to isotopomers, adduct ions, and other product ions of known metabolites were excluded, and remaining peaks were annotated according to the HMT metabolite database based on their *m*/*z* values and MTs. The areas of the annotated peaks were normalized to internal standards and sample amounts to obtain the relative levels of each metabolite. The primary 110 metabolites were quantified based on one-point calibrations using their respective standard compounds. Hierarchical cluster analysis and principal component analysis were performed using proprietary MATLAB and R programs, respectively.^65^ Detected metabolites were plotted on metabolic pathway maps using VANTED software.^66^

### Immunofluorescence and quantification

The cultured IO, InS^exp^, and tissue were fixed with 10% formalin (Sigma-Aldrich) solution overnight and incubated with sucrose (15–30%) for cryopreservation. The samples were frozen in OCT compound (Sakura, Japan). The frozen IO, InS^exp^, and tissue blocks were cut into 10-μm sections, which were permeabilized with 0.1% Triton X-100 (Sigma-Aldrich) containing PBS and blocked with 4% bovine serum albumin (Bovogen Biologicals, Australia) containing PBS for 1 h. The sections were incubated with the appropriate primary antibody overnight at 4°C. After washing with PBS containing 0.05% Tween 20 (Sigma-Aldrich), the sections were incubated with fluorescently labeled secondary antibodies for 1 h. Nuclei were stained with 4’,6-diamidino-2-phenylindole, dihydrochloride (DAPI, Thermo Fisher Scientific). Images were captured using an LSM800 confocal microscope (Carl Zeiss, Gottingen, Germany). Immunofluorescent images were quantified from at least three individual experiments. Nuclear localized proteins, such as Ki67, were directly counted and normalized using DAPI numbers in the field of view. Membrane protein expression (e.g., CD44, ZO-1, and ECAD) was quantified to mean RGB pixel intensity using ImageJ software (n = 5–10 fields from approximately 10 organoids). The fluorescent images were normalized using DAPI intensity. The primary antibodies used in this study are listed in Supplementary Table S5.

### Quantitative RT-PCR

Total RNA was prepared using an RNeasy Kit (Qiagen, Hilden, Germany). cDNA was synthesized from total RNA using the SuperScript IV First Strand Synthesis System (Invitrogen, Carlsbad, CA, USA) according to the manufacturer’s instructions. A 7500 Fast Real-Time PCR system (Applied Biosystems, Foster City, CA, USA) was used for quantitative PCR analysis. The primers used are listed in Supplementary Table S6.

### Acid and bile tolerance; bacterial viability in the GIT

The acid and bile tolerance of the strains was evaluated as described previously.^67^ Lactobacilli strains were grown in MRS broth at 37°C overnight, and sub-cultured (1% v/v) for 3 h at 37°C in 10 mL of fresh MRS broth adjusted to pH 3.0 with 3.0 N HCl, diluted in PBS, and counted for each sample. The survival rate was calculated as the percentage of colony-forming units (CFU) grown on MRS agar compared to the initial bacterial concentration. To determine the bile tolerance, approximately 10^5^ CFU/mL was inoculated into MRS broth supplemented with 3% bile salts (w/v) (OXOID, Basingstoke, UK). Samples were incubated for 24 h at 37°C, and the survival rate was calculated. To investigate whether *L. reuteri* DS0384 physiological condition were examined during simulated passage through the GIT. The saliva, gastric juice, duodenal juice, and bile used in this experiment were prepared as previously described,^68^ and a multi-step *in vitro* digestion model^69^ was developed to simulate the GIT. Briefly, approximately 10^10^ CFU of freshly cultured DS0384 and KCTC3594^T^ cells were suspended in 3 mL saliva (pH 6.8) for 5 min, then 6 mL gastric juice (pH 1.3) was added to each sample and incubated at 37°C for 2 h. Next, 6 mL duodenal juice (pH 8.1), 3 mL bile juice (pH 8.2), and 1 mL 1 M NaHCO_3_ were added, mixed thouroughly, and incubated for an additional 2 h. To test the viability of LAB cells, samples were recovered after both the gastric juice and duodenal mixture incubations, serially diluted with PBS, and plated on MRS agar to count CFUs. The probiotic strain LGG was used as a control. All experiments were performed in an anaerobic chamber under the same conditions.

### Bacterial adhesion to Caco-2 cells

The epithelial intestinal cell line Caco-2 was used for adhesion experiments. The cells were cultured in MEM supplemented with 10% FBS and 1% penicillin-streptomycin at 37°C in an atmosphere of 5% CO_2_ and 95% air. In 24-well tissue culture plate method, the cells were plated at a concentration of 1 × 10^5^ cells/well in 24-well tissue culture plates (Falcon, BD Biosciences). The cell culture medium (2 mL/well) was changed every 2 d and at 24 h before the adhesion assay. Cells in the late post-confluence stage were used for the assays (after 14–16 d in culture, when counted cell number was approximately 5 × 10^5^ cells/well); these cells were completely differentiated. For adhesion assays, late exponential cultures of the bacteria were washed once with PBS and adjusted with MEM media to 1 × 10^8^ cells/mL without antibiotics. The Caco-2 cell monolayer in the 24-well culture plate was washed with fresh MEM, and 1 mL of suspended bacterial cells in MEM was added to the wells. After incubation for 2 h at 37°C in 5% CO_2_ and 95% air, unattached bacteria were removed by washing the Caco-2 cells five times with sterile PBS. After detaching the Caco-2 cells from the wells by incubation with 1 mL of trypsin-EDTA for 5 min, the cells and adhesive bacteria were transferred to a new tube, serially diluted with PBS, and plated on MRS agar to count the CFU.^70^ All experiments were performed at least twice in triplicate.

### Long-term viability test using hIECs

The hPSCs were directly differentiated into hIECs via generation of hIEC progenitors according to a previously reported procedure.^26^ The hIECs progenitors were maintained with hIEC differentiation medium 1 (DMEM/F-12, 1% P/S, 1% HEPES, 1% NEAA, 1% L-glutamine, 1% N2 supplement, 1% B-27 supplement, 2% FBS, 100 ng/mL epithelial growth factor [EGF], 100 ng/mL R-spondin1, and 5 μg/mL insulin [Thermo Fisher Scientific]). To further differentiate functional IECs, 1.34 × 10^5^ hIEC progenitor cells/cm^2^ were seeded onto 1% Matrigel coated transwell plates (Corning) and treated with hIEC differentiation medium 1 containing 10 μM Y-27632. After 2 d, the cells were moved to hIEC differentiation medium 2 (DMEM/F-12, 1% P/S, 1% HEPES, 1% NEAA, 1% L-glutamine, 1% N2 supplement, 1% B-27 supplement, 2% FBS, 100 ng/mL EGF, 2 μM Wnt-C29 [Selleckchem, Houston, TX, USA], 1 mM VPA [Stemgent, Houston, TX, USA]). The medium was changed every other day. After 14 d, the bacterial co-culture experiment was performed under aerobic and anaerobic conditions. All experiments were performed at least two biological replicates.

### Mouse experiments

Three-day-old (P3) C57BL/6 J mice were stabilized with maternal mice for 2 d. Infant mice (P5) were orally administered PBS, strains of *L. reuteri* DS0384 (5 × 10^6^ CFU), or CFS (1:10 diluted CFS from *L. reuteri* DS0384 and KCTC3594^T^) daily for 7 d. On day 8, the mice were euthanized, and the small and large intestines were isolated for analysis. For RNA-seq analysis and confirmation of intestinal colonization (RT-PCR and FISH), postnatal day 3 (P3) C57BL/6 J mice were gavaged four times with CFS from *L. reuteri* DS0384 and KCTC3594^T^ and strains of *L. reuteri* DS0384, respectively, for 12 h and nursed for 12 h. On day 4, the mice were euthanized, and the jejunum and colon were separated for analysis. Adult (5–6 weeks) C57BL/6 J male mice were orally administered 200 μL of PBS and 100 mM NCG daily for 7 d. On day 7, colitis was induced by adding 2% DSS to drinking water for a further 7 d. PBS and NCG groups were orally administered PBS or NCG for 7 d along with DSS. On day 15, all mice were euthanized and the large intestines were isolated for analysis. Animal experiments were performed with the approval of the Institutional Animal Control Committee of the Korea Research Institute of Bioscience and Biotechnology (approval number: KRIBB-AEC-21245).

### Histological analysis

For histological analysis, mouse small intestine and colon tissues were fixed with 10% formalin and embedded in O.C.T. compound for frozen sectioning. As previously reported, 10-μm-thick sections were stained with hematoxylin and eosin and analyzed under a microscope (BX53, Olympus, Tokyo, Japan).^71^ Histological analysis was performed using ImageJ software.

### Mucus staining

AB-PAS staining was performed to verify the function of mucous-secreting goblet cells. Sections (10-μm) from the tissue were stained with AB (Abcam, Cambridge, UK) and PAS (Millipore Sigma, Billerica, MA, USA) staining kits according to the manufacturer’s protocol. The cells were visualized using an Olympus microscope (BX53).

### Extraction of bacterial DNA and RT-PCR

Mouse colon samples were homogenized in cold 50 mM EDTA (pH 8.0) and bacterial DNA was extracted using a Wizard® Genomic DNA Purification Kit (Promega, A1120) according to manufacturer instructions. RT-PCR was performed using purified bacterial DNA as a template. The reaction mixture contained 10 μL 2X H-Star Taq PCR Master Mix (Biofact, HS303-40 h), 50 ng template DNA, and 1 μL (10 pmol) primer; deionized water was added to a final volume of 20 μL. PCR amplification was performed with the C1000 Touch Thermal Cycler (Bio-Rad, C1000 Touch) under the following conditions: preincubation at 95°C for 15 min; 40 cycles of denaturation at 95°C for 20s, annealing at 60°C for 20s, and extension at 72°C for 30s; and a final extension at 72°C for 5 min. Amplicons were separated on SYBR-stained (Invitrogen, S33102) 2.0% agarose gel by electrophoresis at 135 V for 30 min. The signal was detected with the Gel Doc XR System (Bio-Rad, 1708170EDU). The primers used are listed in Supplementary Table S6.

### FISH assay

A Cy3-labeled rRNA probe was developed for FISH and 100% matched to the 16S rRNA sequence for *L. reuteri*.^72,73^ Mouse intestine samples were fixed with 4% PFA at 4°C for 12 h, then treated with ice-cold 96% ethanol (1:1 v/v) for Gram-positive cell fixation. The fixed samples were dehydrated with ethanol (50, 80, and 96%) and dried at 46°C for 3 min. The samples were permeabilized using lysozyme (2 mg/mL) at 37°C for 1 h. After drying at 46°C for 15 min. The tissue samples were treated with a hybridization buffer containing 5 M NaCl, 1 M Tris-HCl, double-distilled water (ddH_2_O), formamide, 10% sodium dodecyl sulfate (SDS), and 5’-Cy3-labeled rRNA probe. Hybridization was performed at 46°C for 2 h.^74^ The tissues were then washed with pre-heated washing buffer containing 5 M NaCl, 1 M Tris-HCl, 0.5 M EDTA, and ddH_2_O at 48°C for 10 min. After rinsing in washing buffer, the samples were immediately dipped in ice-cold ddH_2_O for 2–3 s. The tissues were placed on a slide and examined with an EVOS system (FL Auto 2, Thermo Fisher Scientific, Inc.) and a confocal microscope at 40x magnification (LSM800, Carl Zeiss). The probes used are listed in Supplementary Table S6.

### RNA-seq and data analysis

RNA samples were analyzed using an Agilent 2100 Bioanalyzer system (Agilent Biotechnologies). Only high-quality RNA samples (RNA Integrity Number ≥ 7.5) were prepared for sequencing. Libraries were constructed using Illumina TruSeq library preparation per manufacturer specifications. RNA sequencing was performed on Illumina HiSeq2500 equipment (Illumina, San Diego, CA, USA) using the standard Illumina RNA-Seq protocol with a read length of 2 × 100 bases. The quality of sequence data was evaluated with the NGSQCToolkit v.2.3.3 and the adapters were removed using Cutadapt v.1.18 with default settings. Low quality sequences were also trimmed via Sickle v.1.33 with a Phred quality threshold score of 20. If the trimmed read contained any ambiguous character (such as N) or was less than 50 bp, it was excluded. After preprocessing of raw reads, the clean reads were mapped to the reference genome (GRCm38) using HISAT2 v.2.0.5 with default parameter settings and applying StringTie v.2.1.0 using the reference annotation file to estimate the expression levels of all genes and transcripts. To correct the scale between samples, all data was normalized using log_2_(FPKM+1). The log_2_ transformation values were used for this analysis, and rows with zero expression in all samples were eliminated (Supplementary Table S3). Before analyzing the differentially expressed genes (DEGs), the Pearson correlation method was applied using the “cor” function of R v.4.0.2 to determine the correlation of replicate sets. A hierarchical clustering map and heatmap were generated by Euclidean distance and ward.D2 cluster method using the “heatmap.2” of the gplots package v.3.1.1.

### Statistical analysis

Experiments were repeated at least thrice and the results are expressed as the mean ± standard error. One-way ANOVA and two-tailed *t*-tests were used to analyze the statistical significance of the data. Calculation statistics and graphing were performed using Excel (Microsoft, Redmond, WA, USA) and GraphPad Prism version 7 for Windows (GraphPad, Inc., La Jolla, CA, USA). Statistical significance was set at *p* < .05.

## Supplementary Material

Supplemental Material

## Data Availability

Whole-genome sequences were deposited under Bioproject PRJNA791146 and Biosample SAMN24255923, respectively. The GenBank accession numbers were CP090313 for a single chromosome and CP090314 for the plasmid. All other data that support the findings of this study are available from the corresponding author upon reasonable request. (https://www.ncbi.nlm.nih.gov/data-hub/genome/GCF_021398615.1/).
