# Supplementary material for: Limosilactobacillus reuteri DS0384 promotes intestinal epithelial maturation via the postbiotic effect in human intestinal organoids and infant mice
Source: Gut Microbes. 2022 Sep 21;14(1):2121580. doi: 10.1080/19490976.2022.2121580 (PMC9519030; doi:10.1080/19490976.2022.2121580)
Supplement: Supplemental Material [file KGMI_A_2121580_SM9890.zip › Supplementary Table S2 Viability of L reuteri DS0384 in the gastrointestinal tract.docx]

**Supplementary Table S2.** Viability of *L. reuteri* DS0384 in the gastrointestinal tract. Viability was tested using multiple-step in vitro digestion model and *L. reuteri* KCTC3594T and LGG were used as reference. Initial CFUs of the LAB were 9.4 x 10^9^, 1.7 x 10^10^, and 1.7x10^10^ for KCTC3594, DS0384, and LGG, respectively.

| Digestive juice | KCTC3594^T^ | | DS0384 | | LGG | |
| --- | --- | --- | --- | --- | --- | --- |
|  | Viable cell  (log CFU) | Viability  (%) | Viable cell  (log CFU) | Viability  (%) | Viable cell  (log CFU) | Viability  (%) |
| S+G | 8.95±0.13 | 9.82±3.14 | 9.61±0.06 | 24.90±3.49 | 9.44±0.09 | 17.11±3.46 |
| *p-value*  (*vs* KCTC3594^T^) |  |  | 0.0003 | 0.0020 | 0.0035 | 0.0353 |
| *p-value*  (*vs* LGG) |  |  | 0.0182 | 0.0193 |  |  |
| S+G+D+B | 7.40±0.13 | 0.28±0.09 | 7.27±0.11 | 0.12±0.03 | 7.38±0.03 | 0.15±0.01 |
| *p-value*  (*vs* KCTC3594^T^) |  |  | 0.0757 | 0.0095 | 0.1492 | 0.0149 |
| *p-value*  (*vs* LGG) |  |  | 0.1573 | 0.1949 |  |  |

^1)^ S+G : Saliva + Gastric juice

^2)^ S+G+D+B : Saliva + Gastric juice + Duodenal juice + Bile juice + NaHCO_3_
