# Supplementary material for: Limosilactobacillus reuteri DS0384 promotes intestinal epithelial maturation via the postbiotic effect in human intestinal organoids and infant mice
Source: Gut Microbes. 2022 Sep 21;14(1):2121580. doi: 10.1080/19490976.2022.2121580 (PMC9519030; doi:10.1080/19490976.2022.2121580)
Supplement: Supplemental Material [file KGMI_A_2121580_SM9890.zip › Supplementary Table S4 The Lactic acid bacterial strains used in this study.docx]

**Supplementary Table S4.** The Lactic acid bacterial strains used in this study.

| Strains | Host | Isolation source |
| --- | --- | --- |
| *Bifidobacterium longum* DS0431 | Human | Infant feces |
| *Lactobacillus gasseri* DS0444 | Human | Breast milk |
| *Lactobacillus crispatus* DS3141 | Human | Vaginal mucosa |
| *Lacticaseibacillus rhamnosus* DS0979 | Human | Infant feces |
| *Limosilactobacillus reuteri* DS0384 | Human | Infant feces |
| *Limosilactobacillus reuteri* DS0195 | Swine | Small intestine |
| *Limosilactobacillus reuteri* DS0333 | Human | Infant feces |
| *Limosilactobacillus reuteri* KCTC3594^T^ | Human | Intestine of adult |
