# Supplementary material for: Limosilactobacillus reuteri DS0384 promotes intestinal epithelial maturation via the postbiotic effect in human intestinal organoids and infant mice
Source: Gut Microbes. 2022 Sep 21;14(1):2121580. doi: 10.1080/19490976.2022.2121580 (PMC9519030; doi:10.1080/19490976.2022.2121580)
Supplement: Supplemental Material [file KGMI_A_2121580_SM9890.zip › Supplementary Table S5 Antibodies utilized in this study.docx]

**Supplementary Table S5.** Antibodies utilized in this study

| Antibodies | Catalog No. | Company | Dilution |
| --- | --- | --- | --- |
| anti-E-Cadherin | AF648 | R&D systems | 1:500 for IF* |
| anti-alpha 5 Defensin | ab90802 | abcam | 1:50 for IF |
| anti-OLFM4 | ab85046 | abcam | 1:100 for IF |
| anti-MUC13 | ab124654 | abcam | 1:100 for IF |
| anti-Cytokeratin 20 | ab76126 | abcam | 1:400 for IF |
| anti-Ki67 | AB9260 | Chemicon | 1:200 for IF |
| anti-CD44 | ab6124 | abcam | 1:200 for IF |
| anti-ZO-1 | 61-7300 | Thermo Scientific | 1:50 for IF |

*IF: Immunofluorescence
