# Supplementary material for: Limosilactobacillus reuteri DS0384 promotes intestinal epithelial maturation via the postbiotic effect in human intestinal organoids and infant mice
Source: Gut Microbes. 2022 Sep 21;14(1):2121580. doi: 10.1080/19490976.2022.2121580 (PMC9519030; doi:10.1080/19490976.2022.2121580)
Supplement: Supplemental Material [file KGMI_A_2121580_SM9890.zip › Supplementary Table S6 Primers and a Probe utilized in this study.docx]

**Supplementary Table S6.** Primers and a Probe utilized in this study

| Gene | Primer (Forward) | Primer (Reverse) |
| --- | --- | --- |
| *GAPDH* | GAAGGTGAAGGTCGGAGTC | GAAGATGGTGATGGGATTTC |
| *ASCL2* | CGTGAAGCTGGTGAACTTGG | GGATGTACTCCACGGCTGAG |
| *CASP3* | TCACAGCAAAAGGAGCAGTTT | TCAATGCCACAGTCCAGTTC |
| *CD44* | CCAGAAGGAACAGTGGTTTGGC | ACTGTCCTCTGGGCTTGGTGTT |
| *CDX2* | CTGGAGCTGGAGAAGGAGTTTC | ATTTTAACCTGCCTCTCAGAGAGC |
| *CLAUDIN* | CCGTTGGCATGAAGTGTATG | CATTGACTGGGGTCATAGGG |
| *CREB3L3* | ATCTCCTGTTTGACCGGCAG | GTCGTCAGAGTCGGGGTTTG |
| *DEFA5* | CCTTTGCAGGAAATGGACTC | GGACTCACGGGTAGCACAAC |
| *DPP4* | TGGAAGGTTCTTCTGGGACTG | CAGCTGTAGCATCATCTGTGCC |
| *EPCAM* | TAAGGCCAAGCAGTGCAAC | GCGTTGTGATCTCCTTCTGA |
| *IL-1β* | AATCTGTACCTGTCCTGCGTGTT | TGGGTAATTTTTGGGATCTACACT |
| *IL-6* | CTCCTTCTCCACAAGCGCC | AAGGCAGCAGGCAACACC |
| *IL-8* | AGTTTTTGAAGAGGGCTGAGA | TGCTTGAAGTTTCACTGGCATC |
| *KRT20* | TGGCCTACACAAGCATCTGG | TAACTGGCTGCTGTAACGGG |
| *LCT* | CTGCAGGCCTCAACAAGTCT | GCCAAAAGGCGTCATCTTCA |
| *LGR5* | TGCTCTTCACCAACTGCATC | CTCAGGCTCACCAGATCCTC |
| *LYZ* | AAAACCCCAGGAGCAGTTAAT | CAACCCTCTTTGCACAAGCT |
| *MKI67* | TGACCCTGATGAGAAAGCTCAA | CCCTGAGCAACACTGTCTTTT |
| *MUC13* | CGGATGACTGCCTCAATGGT | AAAGACGCTCCCTTCTGCTC |
| *MUC2* | TGTAGGCATCGCTCTTCTCA | GACACCATCTACCTCACCCG |
| *OLFM4* | ACCTTTCCCGTGGACAGAGT | TGGACATATTCCCTCACTTTGGA |
| *SLC5A1* | GTGCAGTCAGCACAAAGTGG | ATGCACATCCGGAATGGGTT |
| *SOX9* | GGAGAGCGAGGAGGACAAGTTC | TTGAAGATGGCGTTGGGGG |
| *TNFα* | GGAGAAGGGTGACCGACTCA | CTGCCCAGACTCGGCAA |
| *VIL1* | AGCCAGATCACTGCTGAGGT | TGGACAGGTGTTCCTCCTTC |
| *β-actin* | TGTTACCAACTGGGACGACA | GGGGTGTTGAAGGTCTCAAA |
| *Arg2* | TAGGGTAATCCCCTCCCTGC | AGCAAGCCAGCTTCTCGAAT |
| *Treh* | GACTCAGGAGGTGGCTTTCC | CCATAGCGATCCAGAAGCAT |
| *Lyz1* | GCCAAGGTCTACAATCGTTGTGAGTTG | CAGTCAGCCAGCTTGACACCACG |
| *Gip* | GGGAAAGGAGGACAAAGAGG | GAAGCAGGAGCCAAGCAAG |
| *Lct* | TGTCCTAGCCTACAACCTCAAC | AGCGGTCTGTAATGGAAGCA |
| *Kcnj13* | CGGGTCAAAAAGACAAGAGG | CCATCTGAAGTGTGCTGTGG |
| *Slc2a2* | GTCCTACGGCTCTGGCACT | GCAGCGATTTCCTCAAAAGA |
| *All bacteria* | TCCTACGGGAGGCAGCAGT | GGACTACCAGGGTATCTAATCCTGTT |
| *L. reuteri* | CAGACAATCTTTGATTGTTTAG | GCTTGTTGGTTTGGGCTCTTC |
| *L. reuteri_P* | Cy3- CCAGTGAGTGGCGGACGGGTGAG | |
